# Bilateral Bloody Nipple Discharge in a Primigravid Patient: A Benign Presentation Mimicking Pathology

**DOI:** 10.7759/cureus.109511

**Published:** 2026-05-23

**Authors:** Dimitrios Karamanidis, Aikaterini Koufonikola, Berna Baltzi, Noursen Kechagia

**Affiliations:** 1 Obstetrics and Gynecology NHS, University General Hospital of Alexandroupolis, Alexandroupoli, GRC

**Keywords:** benign breast disease, bloody nipple discharge, breast ultrasound, lactation, pregnancy, rusty pipe syndrome

## Abstract

Bloody nipple discharge during pregnancy is a rare clinical finding that often causes significant anxiety for both the patient and the clinician. Bloody nipple discharge usually raises suspicion of malignancy or intraductal papilloma. However, it can occur as a benign, self-limiting physiological process during pregnancy due to increased breast vascularity and epithelial proliferation (rusty pipe syndrome). We report a case of a 31-year-old primigravid woman at 16 weeks’ gestation presenting with bilateral, spontaneous, multi-duct bloody nipple discharge without associated palpable masses or breast pain. Breast ultrasound revealed no pathological findings, demonstrating normal pregnancy-related parenchymal changes without focal lesions, and cytology confirmed benign findings. Cytology played a supportive, not diagnostic, role because of its relatively low sensitivity. The patient was managed conservatively with close clinical surveillance. Symptoms remained stable throughout the gestational period and resolved spontaneously 10 days postpartum. This case highlights the importance of recognizing benign causes of bloody nipple discharge in pregnancy and supports a non-invasive, stepwise diagnostic approach (triple assessment) to avoid unnecessary invasive interventions in the pregnant patient while ensuring patient safety.

## Introduction

Nipple discharge is the third most common breast-related complaint after mastalgia and palpable masses [1]. When the discharge is bloody, the primary concern is the exclusion of breast cancer, which complicates approximately one in 3,000 pregnancies [2]. Pathologic nipple discharge is defined as fluid that occurs spontaneously and is serosanguinous, serous, or watery. Most pathologic nipple discharge is due to a benign etiology such as intraductal papilloma (35%-56% of cases) and duct ectasia (6%-59% of cases). The risk of malignancy ranges from 5%-23% of cases (ductal carcinoma in situ and invasive breast cancer) [3]. Clinical studies estimate the prevalence of physiological bloody discharge in pregnancy at approximately 0.1% of pregnancies [4]. Malignant or papillomatous lesions typically present with unilateral, single-duct discharge. However, bilateral multi-duct bloody nipple discharge in pregnancy is frequently associated with benign physiological changes, often referred to as "rusty pipe syndrome." This phenomenon is thought to result from the rapid growth of the alveolar network and increased vascularization of the breast tissue, leading to the rupture of small capillaries into the ductal system [3]. Rusty pipe syndrome is a specific, benign clinical subset of physiologic discharge, most common in primiparous women, characterized by painless, bilateral, brown-colored milk that mimics water from a rusty pipe. This specific condition typically resolves within seven days of the onset of lactation, and literature indicates it almost always completely disappears within 10 days postpartum [5]. Recognizing this self-limiting condition is crucial to prevent maternal stress and avoid unnecessary surgical interventions during pregnancy, but the exclusion of malignancy remains mandatory.

## Case presentation

A 31-year-old primigravid woman at 16 weeks’ gestation presented with a one-week history of bilateral nipple discharge. The patient reported that the discharge was dark brown and occurred spontaneously, staining her clothing. She denied any associated breast pain, fever, or history of trauma. There was no personal or family history of breast or ovarian cancer, apart from a previously known 1.4 cm fibroadenoma in the left breast at the nine o’clock position.

Clinical breast examination revealed bilateral, non-tender breasts without any palpable masses, skin changes, or axillary lymphadenopathy. Upon manual expression, bloody discharge was noted from multiple duct openings on both nipples.

**Figure 1:**
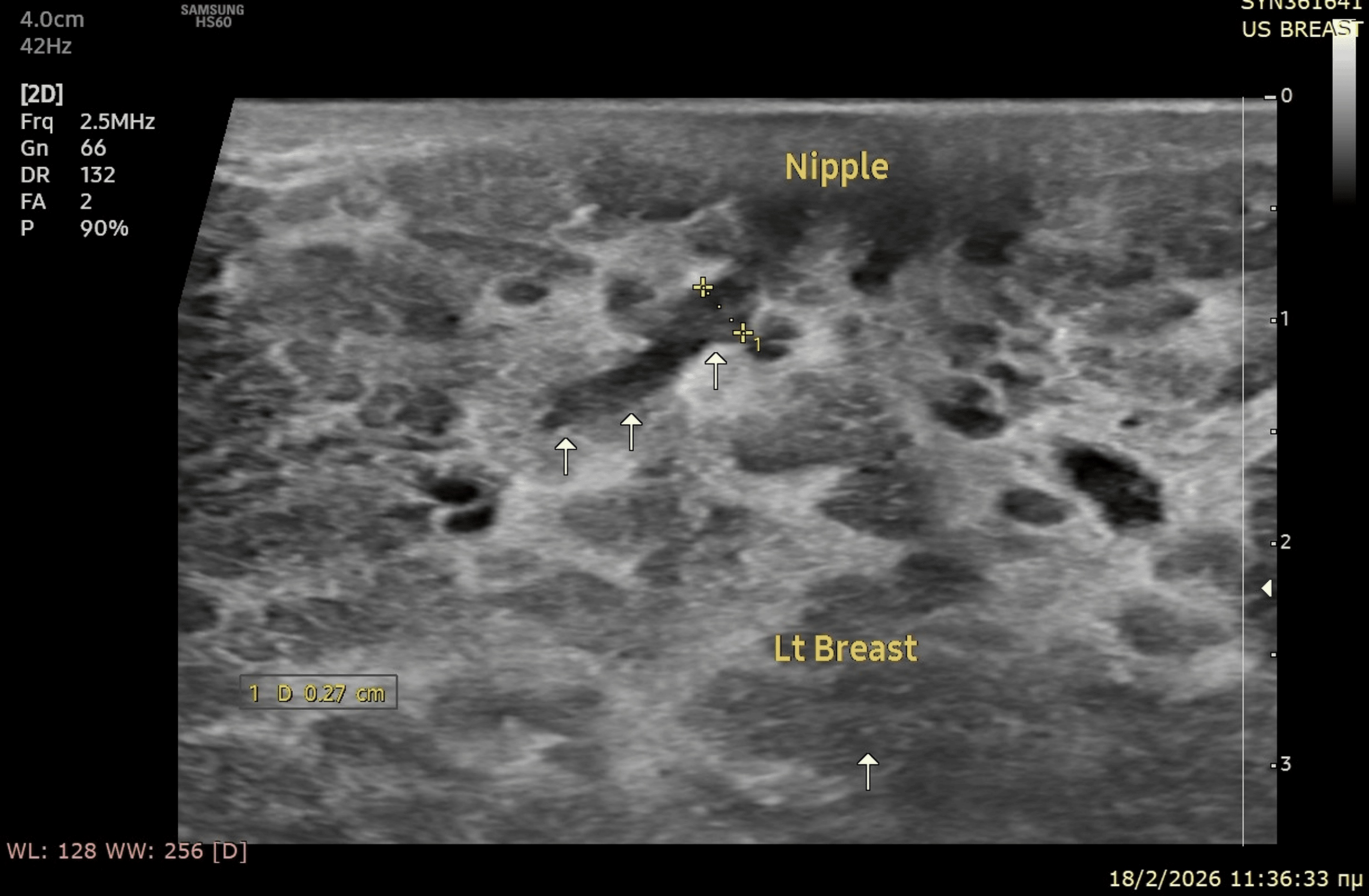
Ultrasound image of left breast: retroareolar region The white arrows point to a prominent subareolar duct (2.7 mm) with low-level internal echoes, representing concentrated fluid mixed with red blood cells. The lumen of the duct appears patent. No sonographic evidence of solid breast masses despite dense fibroglandular parenchyma. The surrounding tissue appears very dense (hyperechoic) with cystic physiological changes of pregnancy.

**Figure 2:**
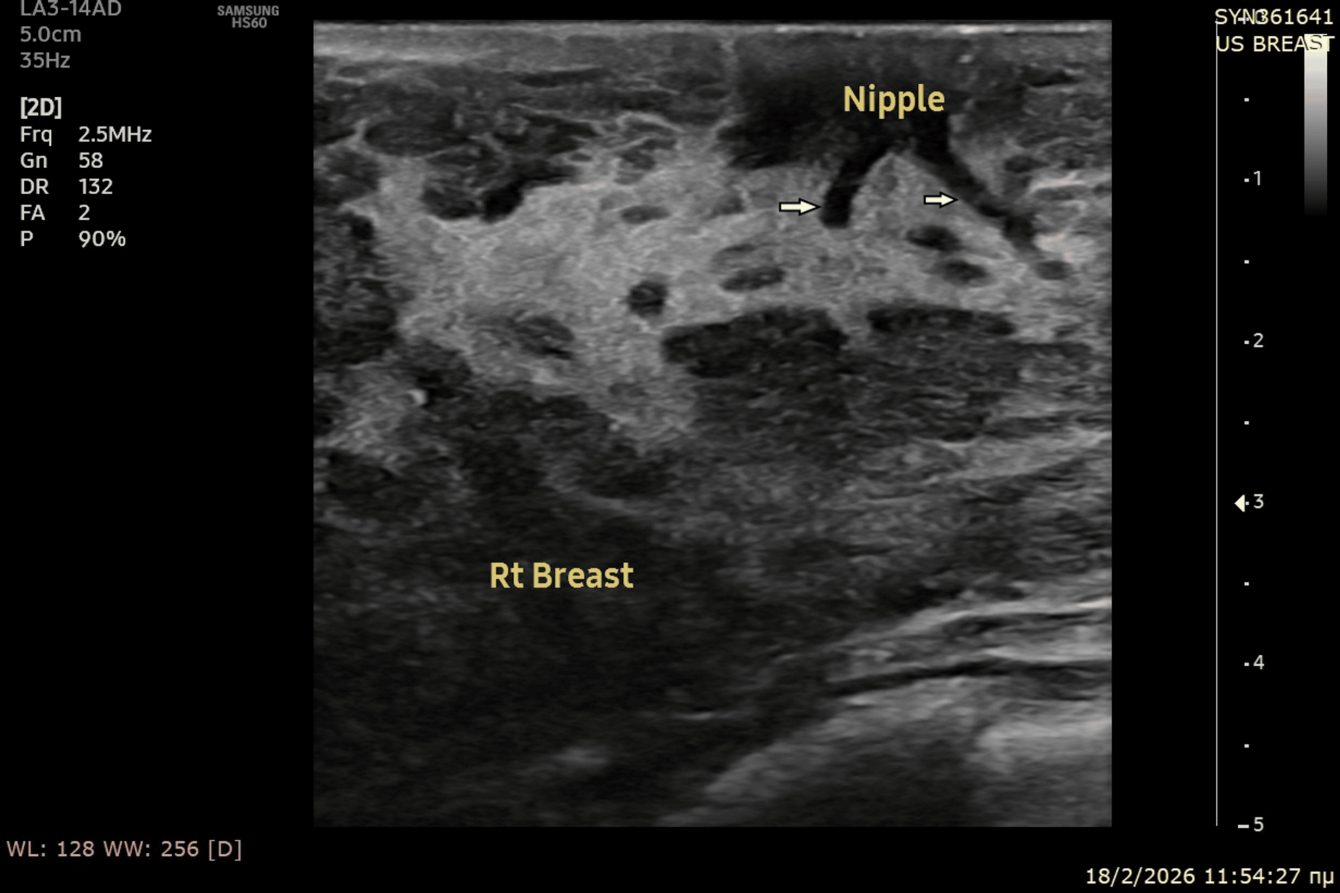
Ultrasound image of right breast: retroareolar region Similar sonographic findings were observed in the right breast, demonstrating physiological changes of pregnancy. White arrows show major ducts.

A bilateral breast ultrasound was performed, which showed physiologic gestational changes of the breast parenchyma without evidence of intraductal lesions, suspicious solid masses, or ductal ectasia. The previously documented benign fibroadenoma of the left breast was again identified: breast imaging reporting and data system (BI-RADS) 2 (Figures 1, 2).

**Figure 3:**
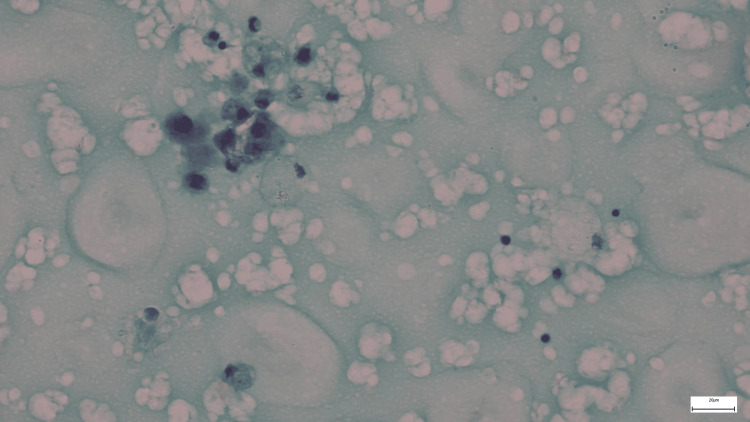
Liquid-based cytology of the discharge The smear exhibits a lipid-rich background characterized by numerous lipid vacuoles. There are cohesive clusters of ductal epithelium (cells with enlarged nuclei but not atypia) and some foamy macrophages (histiocytes). Erythrocytes were cleared during ThinPrep processing as they passed through the filter pores, resulting in a clean background focused on epithelial and gestational cellular changes.

Cytological examination of the discharge was performed to further characterize the findings. The smears revealed the presence of numerous red blood cells, occasional foamy macrophages, a small number of inflammatory leukocytes, and small, cohesive clusters of benign ductal epithelial cells without atypia (Figure 3).

Given the bilateral nature, the origin from multiple duct openings, the absence of a mass on ultrasound, and the benign cytology, a conservative approach was adopted, with close clinical follow-up. The patient was re-evaluated three times during the remainder of her pregnancy (at 24, 32, and 36 weeks). At each visit, clinical examination and ultrasound remained stable, with no appearance of "red flag" symptoms (such as a palpable breast or axillary mass, skin changes, a newly retracted or inverted nipple, or development of infection signs) or new masses detected on ultrasound. The patient reported requiring 3-4 breast pads per day during the third trimester. Following delivery, the patient began breastfeeding. The discharge transitioned from bloody to serosanguinous and ceased completely by the 10th day of the puerperium. A diagnosis of rusty pipe syndrome was made. The patient was informed about the option for further evaluation in the puerperium. Following resolution of the bloody discharge, she declined additional investigation at the present time, stating her preference to focus on breastfeeding and care of her newborn. She acknowledged the medical advice provided and was advised to seek reassessment in case of recurrent symptoms or clinical concern.

## Discussion

Bloody nipple discharge during pregnancy is an alarming symptom that often prompts evaluation for underlying breast pathology. While unilateral discharge is more commonly associated with pathological conditions, bilateral discharge, particularly in pregnancy, may suggest a benign etiology. During pregnancy, hormonal stimulation leads to rapid proliferation of the ductal epithelium and the supporting stroma, accompanied by increased capillary fragility, leading to micro-extravasation of blood into the ductal lumen [4]. This condition, often referred to as “rusty pipe syndrome,” is typically self-limiting and resolves spontaneously, usually during late pregnancy or early postpartum [5].

The primary differential diagnosis for pathological bloody nipple discharge in pregnancy includes intraductal papilloma (usually presents as unilateral, single-duct discharge), pregnancy-associated breast cancer (often presents with a palpable mass; however, pathological bloody nipple discharge can be a presenting symptom), and mastitis (usually accompanied by pain, erythema, and fever). The "triple assessment" (clinical exam, imaging, and cytology/pathology) remains the gold standard. In pregnant patients, ultrasound is the first-line imaging modality due to its high sensitivity in dense breast tissue and lack of ionizing radiation [1,3,6,7].

In this case, the bilateral and multi-ductal nature of the discharge was highly suggestive of a systemic physiological process rather than a localized lesion. Cytology played a supportive role; the presence of foamy cells and the absence of high-grade nuclear atypia provided reassurance. The reported sensitivity of nipple discharge cytology is relatively low (approximately 11%-34%), whereas specificity is generally higher (approximately 81%-100%); therefore, cytology alone cannot reliably exclude underlying malignancy. Although the diagnostic value of nipple discharge cytology is limited, it may provide supportive information in selected pregnant patients because it is inexpensive, non-invasive, and easily repeatable. However, cytologic findings should always be interpreted in conjunction with clinical examination and imaging studies.

The management of bilateral pathological bloody nipple discharge in pregnancy is primarily observational. Most literature suggests that if imaging is negative, the patient can be monitored throughout the pregnancy. Intervention is only indicated if a suspicious mass develops or if the symptoms do not resolve within a few weeks postpartum [1,3,5,8]. In our patient, the resolution 10 days after delivery confirms the transient nature of the condition. While most bloody nipple discharge during pregnancy is physiologic and self-limiting, the following scenarios require escalation to diagnostic workup: (1) Failure to resolve within seven to 10 days postpartum. Persistence beyond these timeframes warrants investigation via breast ultrasound and potentially mammogram and breast MRI. (2) Appearance of "red flag" symptoms such as a palpable breast or axillary mass, skin changes, a newly retracted or inverted nipple, or unilateral discharge suggesting papilloma or malignancy. (3) Development of infection signs.

Even after symptoms resolve, sources recommend structured follow-up to ensure long-term stability with an initial reassessment two months postpartum to confirm complete resolution and a follow-up period of up to two years with clinical examination, ultrasound every six months, and potentially annual mammography [4]. This case underscores the importance of distinguishing physiological from pathological causes to reduce unnecessary biopsies and diagnostic imaging while ensuring maternal safety.

## Conclusions

Bilateral bloody nipple discharge at 16 weeks’ gestation, in the absence of clinical, imaging, or cytological suspicion for malignancy, is most likely a benign physiological event. Clinicians should maintain a high index of suspicion for malignancy but must balance this with the knowledge that bilateral bloody nipple discharge in pregnancy and lactation is a self-limiting condition. A thorough diagnostic workup with ultrasound and cytology is essential for patient reassurance. If the workup is negative, conservative management and clinical monitoring are appropriate, as symptoms typically resolve shortly after the onset of lactation. Recognition of this entity is important to prevent unnecessary diagnostic or surgical interventions.
